# Ground Glass Opacity and Adjuvant Chemotherapy in Pathological Stage IB–IIA Lung Adenocarcinoma

**DOI:** 10.3389/fonc.2022.851276

**Published:** 2022-03-25

**Authors:** Wenyu Zhai, Li Gong, Yuzhen Zheng, Qihang Yan, Renchun Lai, Dachuan Liang, Wingshing Wong, Shuqin Dai, Junye Wang

**Affiliations:** ^1^ Department of Thoracic Surgery, State Key Laboratory of Oncology in South China, Collaborative Innovation Center for Cancer Medicine, Sun Yat-sen University Cancer Center, Guangzhou, China; ^2^ Department of Thoracic Surgery, The Second Department of Surgery, Sun Yat-sen University Sixth Affiliated Hospital, Guangzhou, China; ^3^ Department of Thoracic Surgery, The Second Affiliated Hospital Zhejiang University School of Medicine, Hangzhou, China; ^4^ Department of Anaesthesiology, State Key Laboratory of Oncology in South China, Collaborative Innovation Center for Cancer Medicine, Sun Yat-sen University Cancer Center, Guangzhou, China; ^5^ Department of Laboratory Medicine, State Key Laboratory of Oncology in South China, Collaborative Innovation Center for Cancer Medicine, Sun Yat-sen University Cancer Center, Guangzhou, China

**Keywords:** lung adenocarcinoma, ground glass opacity, adjuvant chemotherapy, nomogram, personalized therapy

## Abstract

**Background:**

The prognostic value of ground glass opacity (GGO) in stage IA non-small cell lung cancer (NSCLC) has been widely recognized. However, studies investigating its value in the related stage IB–IIA lung adenocarcinoma (LUAD) remains lacking. The impact of adjuvant chemotherapy (ACT) on pathological stage IB–IIA LUAD is also controversial.

**Materials and Methods:**

We retrospectively reviewed the clinical records of 501 patients with pathological stage IB–IIA LUAD at the Sun Yat-sen University Cancer Center from January 2008 to June 2018. We calculated and compared survival curves using the Kaplan–Meier test and log-rank test. Cox regression models were performed to determine independent prognostic factors of disease-free survival (DFS) and overall survival (OS). We established nomograms to predict the OS and DFS of LUAD patients. Calibration and receiver operator characteristic curves were conducted to assess the predictive performance of two nomograms. Based on the nomogram, we identified candidate patients that may most benefit from ACT after surgery.

**Results:**

The number of patients with pure solid, part GGO, and pure GGO nodules was 240, 242, and 19, respectively, and 125 patients who received ACT. Patients with consolidation-to-tumor ratio (CTR) <0.75 had longer OS (*P* = 0.026) and DFS (*P* = 0.003). Pathological tumor size and at least 10 lymph nodes (LNs) resection were independent prognostic factors of both OS and DFS. CTR <0.75 was positively associated with DFS. The C-index of nomograms predicting individual OS and DFS was 0.660 and 0.634, respectively. Based on the nomogram for OS, ACT was found to be a positive prognostic indicator of OS (*P* = 0.031, HR = 0.5141, 95% CI 0.281–0.942) in patients with nomogram total points ≥5.

**Conclusion:**

CTR <0.75 is associated with a better DFS in patients with stage IB–IIA LUAD. Nomograms developed by integrating pathological tumor size, at least 10 LNs resection, and CTR ≥0.75 for predicting individual OS and DFS displayed a good predictive capacity and clinical value, which were also proved to be a useful tool for selecting patients most benefiting from ACT.

## Introduction

Lung cancer is the second most common malignancy with the highest mortality worldwide (1). More than 40% of lung cancer patients are diagnosed with lung adenocarcinoma (LUAD), which is the major histological type of all lung cancer types (2, 3). Radical resection is the cornerstone therapy for patients with stage IB–IIA non-small cell lung cancer (NSCLC) (4), but the prognosis remains unsatisfactory due to a relatively high rate of recurrence (5, 6). The 5-year overall survival (OS) rate of pathological stage IB–IIA NSCLC patients ranges from 74.6 to 84.6% (7), suggesting the highly heterogeneous of stage IB–IIA (T2a–2bN0M0) NSCLC, thus, individualized treatment based on risk stratification of patients using multiple prognostic indicators may be a better treatment strategy.

Ground glass opacity (GGO) has been widely considered as a prognostic factor for LUAD patients (8–10). However, most studies on GGO have focused only on patients with stage IA LUAD (10–12), few studies have included a small proportion of stage IB LUADs (13, 14). To date, no study has been specifically designed for stage IB–IIA LUAD to explore the prognostic value of GGO.

Several large randomized controlled trials (RCT) have shown that postoperative adjuvant chemotherapy (ACT) is associated with improvement in survival for patients with lymph node metastasis (15–17), but the role of ACT in patients with stage IB–IIA remains controversial. The CALGB 9633 trial, the only multi-center RCT specially designed for pathological stage IB (7th American Joint Committee on Cancer (AJCC) criteria), failed to report ACT improved OS (18). Considering the heterogeneity of patients with stage IB–IIA NSCLC, despite the identified ineffectiveness of ACT in improving OS, personalized therapy to identify subpopulations that may benefit from ACT is worth exploring. The National Comprehensive Cancer Network (NCCN) guidelines (Version 6. 2021) defined six main high-risk factors, namely, tumors >4 cm, poorly differentiated tumors, visceral pleural involvement (VPI), vascular invasion (VI), unknown lymph node status, and wedge resection for stage IB–IIA patients. These factors may be considered at the time of deciding treatment with adjuvant chemotherapy. One previous study reviewed 2,633 stage I NSCLC patients and found that ACT improved survival in patients with VI (19). Another study using the data from the Surveillance, Epidemiology and End Results (SEER) database discovered that stage IB patients with poor differentiation can benefit from ACT (20).

Although nomograms, namely, sex, age, genetic polymorphism, number of lymph nodes resected, and degree of differentiation for stage IB NSCLC have been reported (20, 21), a nomogram integrating GGO component to predict individual survival for identifying stage IB–IIA LUAD patients that may benefit from ACT remains lacking. Therefore, in this study we investigated the prognostic value of GGO components in patients with pathological stage IB–IIA LUAD and constructed a corresponding nomogram. Based on this nomogram, we identified the candidate subgroup that may benefit from ACT.

## Materials and Methods

### Patients

Patients pathologically diagnosed with stage IB to IIA LUAD under the 8th edition of the AJCC staging system who underwent radical surgery from January 2008 to March 2018 at the Sun Yat-sen University Cancer Center (SYSUCC) were enrolled in this study. The study was approved by the Institutional Review Board of SYSUCC. Patients who met the following criteria were included (1): pathological diagnosis of stage IB–IIA NSCLC (2); confirmed negative surgical margin (R0); and (3) preoperative thin-section computed tomography (CT) scan for GGO measure and radiological evaluation. The key exclusion criteria were as follows (1): received neoadjuvant therapy (2); multiple primary tumor (3); death within 1 month after surgery; and (4) intolerability to chemotherapy.

### Radiological Evaluation and Definition of CTR

The findings of preoperative thin-section CT of all patients were reviewed by two radiologists independently to distinguish GGO and pure-solid tumor. Controversies were resolved through discussion or an adjudicating senior radiologist. The maximum diameter of the whole tumor in the lung window (a window level of −400 H and a window width of 1,400 H) was defined as whole tumor size, while the maximum diameter of the solid component of the tumor in lung window was defined as solid tumor size. The average of observed whole tumor size and solid tumor size from two radiologists were used as whole tumor size and solid tumor size. We calculated the consolidation tumor ratio (CTR) as the ratio of the solid tumor size to the whole tumor size. According to the definition of GGO, the CTR of pure-GGO is 0, the CTR of part-GGO ranges from 0 to 1, and the CTR of pure-solid is 1.

### Adjuvant Chemotherapy

The ACT regimens for patients were based on the NCCN guidelines and performed personal adjustments by medical oncologist or thoracic surgical oncologist, namely, platinum-based doublet chemotherapy, single platinum chemotherapy, or single pemetrexed chemotherapy. The details of the regimen selection for ACT were determined by a thoracic surgical oncologist or a medical oncologist. ACT was recommended within 4 months of surgery.

### Follow-up and Endpoints

All of the patients underwent follow-up every 3 months for the first 2 years, every 6 months until 5 years, and once a year in subsequent years with routine blood tests, blood biochemical workups, serum biomarkers of lung cancer, and chest and epigastric CT. Positron emission tomography, bone scintigraphy, and brain magnetic resonance imaging were not routine examinations, but were performed if needed.

The main endpoints of this study were OS and disease-free survival time (DFS). OS was calculated from the date of the surgical procedure to the date of the last follow-up or death from any cause. DFS was defined as the time interval between the date of operation to the date of the first event recurrence or death from any cause.

### Statistical Analysis and Construction of the Nomogram

Continuous variables were presented as the mean ± SD and compared using Student’s t-test or Mann–Whitney U test. Categorical variables were tested using the Chi-square test. OS and DFS were estimated using the Kaplan–Meier method and differences between stratifications were compared with log-rank tests. Univariate Cox regression analysis was used to identify the prognostic factors with a *P <*0.05, which were further analyzed using multivariate Cox proportional hazards regression models. All factors with a *P <*0.05 in multivariate analysis of OS or DFS were selected to construct the prognostic nomogram using the R package “rms”. We calculated the concordance index (C-index) to evaluate the predictive accuracy of this prognostic nomogram and generated calibration curves to present the association between predictive survival in our nomograms and actually observed outcomes.

All statistical analyses were performed using R software (version 4.0.3; http://www.r-project.org) and SPSS software version 22.0 for Windows (SPSS Inc., Chicago, IL, USA). A two-sided, *P <*0.05 was considered significant.

## Results

### Patient Characteristics

The baseline characteristics of 501 LUAD patients are shown in **Table 1**. There were 240 patients with pure solid nodules and 261 patients had nodules with GGO component, including 19 with pure GGO nodules. The median age of the 253 male and 248 female patients is 61 years old (ranging from 30 to 82). Compared to patients with pure solid nodules, patients with GGO nodules had a higher proportion in the IB stage (*P* = 0.038) and high differentiation degree (*P* = 0.017), while other baseline characteristics were well balanced.

**Table 1:** Patient’s characteristics.

Characteristics	Pure solid nodulesn = 240	GGO nodulesn = 261	P-value
Gender			0.299
Male	127 (52.9)	126 (48.3)	
Female	113 (47.1)	135 (51.7)	
Age (year)	60.7 ± 9.1	59.9 ± 9.0	0.294
Pathological tumor size (cm)	2.9 ± 1.0	2.7 ± 0.9	0.072
Smoking history			0.277
No	146 (60.8)	171 (65.5)	
Yes, or ever	94 (39.2)	90 (34.5)	
8^th^ TNM stage			**0.038**
IB	208 (86.7)	241 (92.3)	
IIA	32 (13.3)	56 (7.7)	
Differentiation degree			**0.016**
Well	8 (3.3)	27 (10.3)	
Moderate	140 (58.3)	159 (60.9)	
Poor	92 (38.3)	75 (28.7)	
Visceral pleura invasion			0.264
Positive	159 (66.3)	185 (70.9)	
Negative	81 (33.8)	76 (29.1)	
Vascular invasion			0.669
Positive	30 (12.5)	36 (13.8)	
Negative	210 (97.5)	225 (86.2)	
Adjuvant chemotherapy			0.817
Positive	61 (25.4)	64 (24.5)	
Negative	179 (74.6)	197 (75.5)	
Operative approach			0.962
Sublobectomy	9 (3.8)	10 (3.8)	
Standard or extended lobectomy	231 (96.2)	266 (96.2)	
Number of LNs examination	21.6 ± 11.0	20.9 ± 11.1	0.506
Thoracotomy or VATS			0.076
Thoracotomy	106 (44.2)	136 (52.1)	
VATS	134 (55.8)	125 (47.9)	

The bold values represented statistically significant.

Among the total 501 LUAD patients in this study, 125 received ACT. Pemetrexed plus carboplatin (n = 51; 40.8%) was the most commonly used regimen. Other used regimens included pemetrexed plus cisplatin (n = 32; 25.6%) and pemetrexed plus nedaplatin (n = 15; 12.0%). Rare regimens included paclitaxel plus cisplatin, paclitaxel plus carboplatin, and gemcitabine plus cisplatin. Besides, 21 patients had a single-agent regimen, such as pemetrexed (n = 18; 14.4%) or cisplatin (n = 3; 2.4%). The details of ACT regimen are shown in **Supplementary Table 1**.

### Survival Analysis

**Figure 1:**
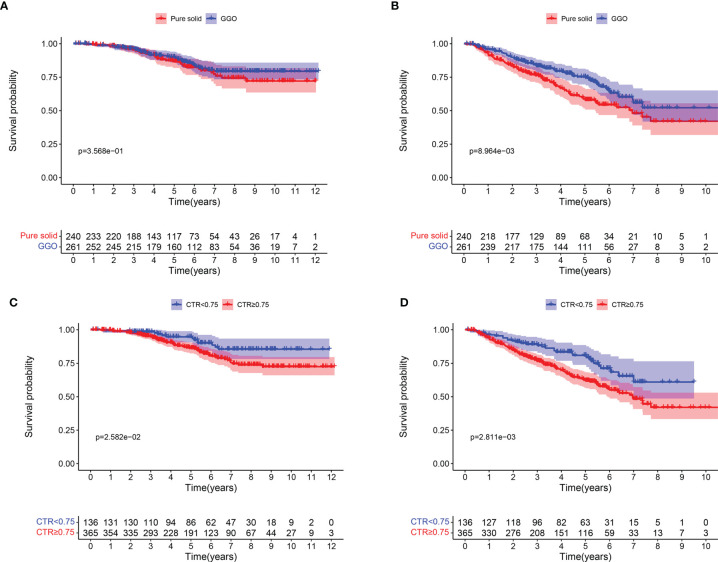
Survival curves for patients with pure solid nodule and GGO nodules. **(A)** Overall survival; **(B)** Disease-free survival. Survival curves for patients with CTR <0.75 AND CTR ≥0.75. **(C)** Overall survival; **(D)** Disease-free survival.

The median overall follow-up time was 64.8 month. There was no apparent difference in 5-year OS rate between GGO and pure solid patients (90.0% vs 86.8%, log-rank *P* = 0.357; **Figure 1A**), though patients with GGO component had a significantly better 5-year DFS rate than those with pure solid patients (75.2% vs 58.9%, log-rank *P* = 0.009; **Figure 1B**). We divided patients into two subgroups according to their CTR value (CTR <0.75 and CTR ≥0.75). Patients with CTR <0.75 showed longer 5-year OS rate (94.7% vs 86.3%, log-rank *P* = 0.026; **Figure 1C**) and 5-year DFS rate (80.8% vs 62.5%, log-rank *P* = 0.003; **Figure 1D**) than those with CTR ≥0.75.

As shown in **Table 2**, gender (*P* = 0.039), pathological tumor size (*P* = 0.002), smoking history (*P* = 0.016), 8th TNM stage (*P* = 0.007), at least 10 lymph nodes (LNs) resection (*P* = 0.007) and CTR ≥0.75 (*P* = 0.029) were identified as significant factors in the univariate analysis of OS and were further carried forward for multivariate analysis, which showed that pathological tumor size (*P* = 0.003, HR 1.408, 95% CI 1.126–1.760), and at least 10 LNs resection (*P* = 0.008, HR 0.459 95% CI 0.260–0.813) were statistically significant for OS for LUAD patients.

**Table 2:** Univariate and multivariate analyses for all patients.

Factors	Univariate Analysis	Multivariate Analysis	
	P value	HR (95% CI)	P-value
**Analysis of OS**			
Gender	**0.039**	0.874 (0.440–1.742)	0.704
Age (year)	0.061		
Pathological tumor size (cm)	**0.002**	1.408 (1.126–1.760)	**0.003**
Smoking history	**0.016**	1.517 (0.953–2.416)	0.079
8th TNM stage (IB versus IIA)	**0.007**	1.170 (0.516–2.655)	0.707
Differentiation degree			
Well	Reference		
Moderate	0.892		
Poor	0.324		
Visceral pleura invasion	0.299		
Vascular invasion	0.384		
Operative approach	0.573		
Adjuvant chemotherapy	0.092		
At least 10 LNs resection	**0.007**	0.459 (0.260–0.813)	**0.008**
GGO component			
CTR <0.75	Reference		Reference
CTR ≥0.75	**0.029**	1.756 (0.877–3.131)	0.120
Thoracotomy or VATS	0.643		
**Analysis of DFS**			
Gender	**0.038**	0.858 (0.619–1.189)	0.358
Age (year)	0.473		
Pathological tumor size (cm)	**<0.001**	1.275 (1.084–1.500)	**0.003**
Smoking history	0.185		
8th TNM stage (IB versus IIA)	**0.024**	0.911 (0.508–1.632)	0.753
Differentiation degree			
Well	Reference		Reference
Moderate	0.216	1.402 (0.673–2.917)	0.374
Poor	**0.033**	1.760 (0.828–3.742)	0.142
Visceral pleura invasion	0.912		
Vascular invasion	0.774		
Operative approach	0.250		
Adjuvant chemotherapy	0.719		
At least 10 LNs resection	**0.005**	0.523 (0.347–0.789)	**0.002**
GGO component			
CTR <0.75	Reference		Reference
CTR ≥0.75	**0.003**	1.582 (1.045–2.393)	**0.030**
Thoracotomy or VATS	0.935		

OS, overall survival' DFS, disease-free survival; VATS, Video-assisted Thoracoscopic Surgery; GGO, ground-glass opacity; CTR, consolidation-to-tumor ratio; LN, lymph node. The bold values represented statistically significant.

The results of survival analysis of DFS were also shown in **Table 2**. In univariate analysis, gender (*P* = 0.038), pathological tumor size (*P <*0.001), 8th TNM stage (*P* = 0.024), poor differentiation degree (*P* = 0.033), at least 10 LNs resection (*P* = 0.005) and CTR ≥0.75 (*P* = 0.003) were statistically significant. After adjusting for other factors in multivariate Cox regression analysis, pathological tumor size (*P* = 0.003, HR 1.275; 95% CI 1.084–1.500) and CTR ≥0.75 (*P* = 0.030, HR 1.582 95% CI 1.045–2.393) were negative prognostic factors of DFS, while at least 10 LNs resection (*P* = 0.002, HR 0.523, 95% CI 0.347–0.789) was positively associated with DFS.

### Development of the Prognostic Nomograms

**Figure 2:**
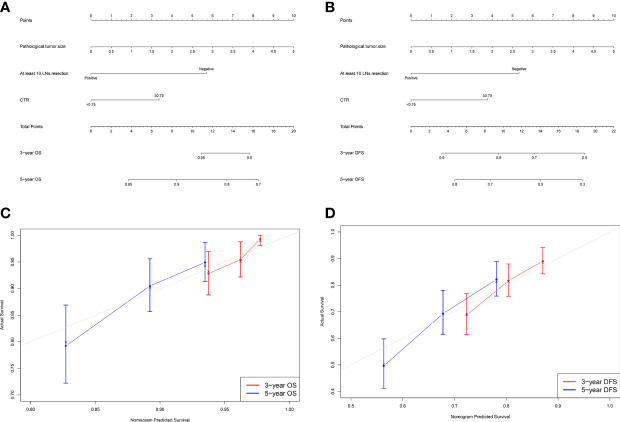
Nomogram for predicting the 3-year and 5-year survival rates in patients with stage IB–IIA LUAD. **(A)** Overall survival (OS); **(B)** Disease-free survival (DFS). For each patient, the points of the three factors are represented as points by projecting them onto the uppermost line (point scale). For panel **(A)**, patients with less than 10 LNs resection get 6 points, otherwise 0 point; patients with CTR ≥0.75 get 3 points, otherwise 0 point. For panel **(B)**, patients with less than 10 LNs resection get 5 points, otherwise 0 point; patients with CTR ≥0.75 get 4 points, otherwise 0 point. For panels **(A, B)**, multiply tumor size by two is the tumor size point of each patient. Totaling the points of three variables is the total point, and projecting the total point value downward onto the bottommost line can determine the probability of 3- and 5-year OS and DFS. Calibration curves for predicting the 3- and 5-year survival rates in stage IB–IIA LUADs. **(C)** OS; **(D)** DFS. The x-axis represents the predicted probability of survival, the y-axis represents the actual probability of survival, and the ideal line is the diagonal of the graph. The closer that the drawn line is to the diagonal, the better is the calibration model. N = 501; The error bars indicate 95% CIs of actual survival.

Based on results of multivariate Cox analyses, we integrated independent indicators, namely, pathological tumor size, at least 10 LNs resection, and CTR ≥0.75, to develop nomograms to estimate the probability of 3- and 5-year OS and DFS (**Figures 2A, B**). The discriminatory performance of the nomograms was evaluated by calculating Harrell’s concordance index (C-index), which was 0.660 (95% CI:0.600–0.721) for OS and 0.634 (95% CI:0.588–0.681) for DFS.

**Figure 3:**
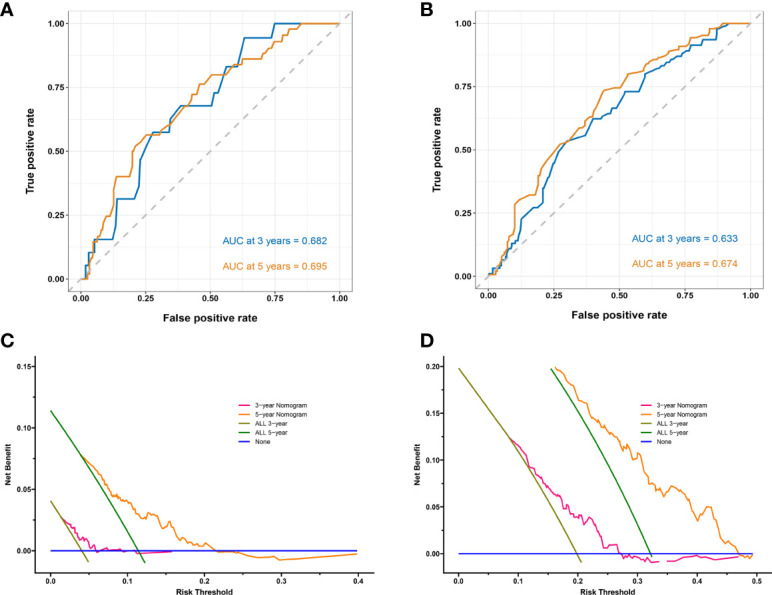
Time receiver operating characteristic (ROC) curves and area under the curve (AUC) for predicting 3-year and 5-year survival rates in stage IB–IIA LUADs. **(A)** Overall survival (OS); **(B)** Disease-free survival (DFS). Decision curves for predicting 3- and 5-year survival rates in stage IB–IIA LUADs. **(C)** OS; **(D)** DFS. The x-axis represents the threshold probabilities and the y-axis measures the net benefit calculated by adding the true positives and subtracting the false positives. The blue line assumes that death or recurrence occurred in no patients. The brown line assumes that all patients will face death or recurrence at a specific threshold probability in 3 years. The green line assumes that all patients will face death or recurrence at a specific threshold probability in 5 years. The pink line represents the net benefit of using the nomogram in 3 years. The yellow line represents the net benefit of using the nomogram in 5 years.

**Figures 2C, D** show the calibration curves of OS and DFS, which suggested a favorable association between the nomogram predictions and the observed OS and DFS at 3- and 5-year. The time receiver operating characteristic (ROC) curve of OS and DFS at 3 and 5 years also indicated the good performance of our nomograms. The area under the curve (AUC) values of the 3- and 5-year OS were 0.682, and 0.695, respectively (**Figure 3A**). The AUC values of the 3- and 5-year DFS were 0.633, and 0.674, respectively (**Figure 3B**).

Decision Curve Analysis (DCA) can be used to evaluate the potential clinical effect of a clinical model. The DCA of these two models demonstrated that net benefit can be achieved for nomograms (**Figures 3C, D**). For the convenient use of these two nomograms, the point for each risk factor in the nomogram and the survival probability associated with the different nomogram total points are provided in **Supplementary Table 2**.

### Identifying Patients That may Benefit From ACT Based on Nomograms

**Figure 4:**
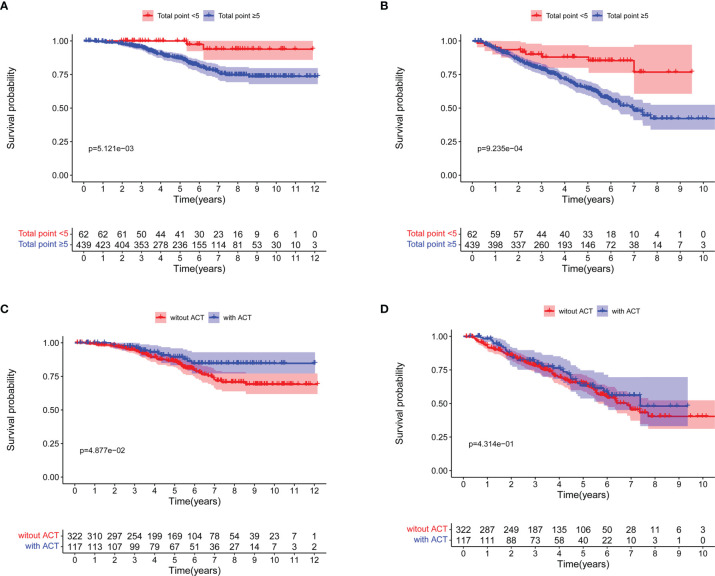
Survival curves for patients with total points <5 and total points ≥5 based on the nomogram for overall survival (OS). **(A)** OS; **(B)** Disease-free survival (DFS). Survival curves for patients with total points ≥5 based on the nomogram for OS. **(C)** OS; **(D)** DFS.

Based on the prognostic nomogram for OS, we could identify patients that may benefit from ACT after surgery. We discovered that 439 patients with nomogram total points ≥5 had worse 5-year OS rate (100.0% vs 87.0%, log-rank *P* = 0.005; **Figure 4A**) and 5-year DFS rate (88.0% vs 64.6%, log-rank *P <*0.001; **Figure 4B**). Moreover, patients with nomogram total points ≥5 who received ACT had a better 5-year OS rate (89.4% vs 86.1%, log-rank *P* = 0.049; **Figure 4C**), but not significant difference was seen in 5-year DFS rate (63.3% vs 65.1%, log-rank *P* = 0.431; **Figure 4D**).

**Table 3** shows the Cox regression analysis of OS for patients with nomogram total points ≥5, from which six statistically significant factors in univariate analysis, that is, pathological tumor size (p = 0.040), smoking history (p = 0.040), 8th edition TNM stage (p = 0.028), visceral pleura invasion (p = 0.020), adjuvant chemotherapy (ACT) (p = 0.049) and at least 10 LNs resection (p = 0.026) were identified. In multivariate analysis, pathological tumor size (*P* = 0.045, HR =1.461, 95% CI 1.009–2.116), visceral pleura invasion (*P* = 0.007, HR = 2.303, 95% CI 1.252–4.238), adjuvant chemotherapy (*P* = 0.031, HR = 0.514, 95% CI 0.281–0.942) and at least 10 LNs resection (*P* = 0.014, HR = 0.475; 95% CI 0.262–0.862) were statistically significant.

**Table 3:** Univariate and multivariate analyses for patients with risk score ≥5.

Factors	Univariate Analysis	Multivariate Analysis	
	P-value	HR (95% CI)	P-value
**Analysis of OS**			
Gender	0.082		
Age (year)	0.052		
Pathological tumor size (cm)	**0.040**	1.461 (1.009–2.116)	**0.045**
Smoking history	**0.040**	1.418 (0.883–2.276)	0.148
8^th^ TNM stage (IB versus IIA)	**0.028**	1.199 (0.516–2.788)	0.673
Differentiation degree			
Well	Reference		
Moderate	0.892		
Poor	0.543		
Visceral pleura invasion	**0.020**	2.303 (1.252–4.238)	**0.007**
Vascular invasion	0.756		
Operative approach	0.573		
Adjuvant chemotherapy	**0.049**	0.514 (0.281–0.942)	**0.031**
At least 10 LNs resection	**0.026**	0.475 (0.262–0.862)	**0.014**
GGO component			
CTR <0.75	Reference		
CTR ≥0.75	0.568		
Thoracotomy or VATS	0.583		

OS, overall survival DFS; disease-free survival; VATS, Video-assisted Thoracoscopic Surgery; GGO, ground-glass opacity; CTR, consolidation-to-tumor ratio. The bold values represented statistically significant.

## Discussion

Though GGO components as a positive prognostic factor in patients with stage IA LUAD (10–12) have been well reported, the evidence of GGO components in stage IB–IIA LUAD is still lacking. In this study, we assessed the prognostic value of GGO in stage IB–IIA LUAD patients and developed nomograms to predict individual survival. In addition, we also identified the subpopulations that may benefit from ACT after surgery based on our nomograms.

There exists substantial evidence on the association of GGO component with prolonged survival in early-stage LUAD patients even a small proportion of GGO components (10), but the association of GGO component with stage IB–IIA LUADs has rarely been investigated (10–12), only a few studies enrolled some patients with stage IB NSCLC. Wang et al. investigated 2,775 patients with pathological stage I NSCLC, wherein, there were1,336 stage IB patients, and found that the presence of GGO was the positively prognostic factor of OS (13). Fu et al. retrospectively reviewed 2,020 patients with pathological stage I NSCLC and included 577 stage IB patients in their cohort. They also revealed that GGO is a positive prognostic factor of recurrence-free survival (14). It should be noted that aforementioned studies included not only massive stage IA patients but also massive lung squamous cell carcinoma (LUSC) patients. It is well-known that nearly all LUSCs appear as pure-solid lesions in CT, which might generate an inevitable bias for results of survival analyses. To the best of our knowledge, this study is the first one specially designed for stage IB–IIA LUADs.

Although previous study has reported that clinical stage IA LUAD patients might benefit from a small proportion of GGO components (10), we found patients with GGO and pure solid nodules had similar OS in current study. We surmise that small proportion of GGO components fails to have a apparent impact on survival in stage IB–IIA LUAD patients. To further explore the prognostic value of CTR, we determined a cutoff value of 0.75 for CTR to stratify patients according to the exploration of Xi et al. (22). In this study, we also found that patients with GGO component and CTR <0.75 had better OS than patients with CTR ≥0.75. Therefore, we used CTR <0.75 instead of GGO component to construct prognostic nomograms.

In the 8th AJCC Staging Manual, patients with tumor size 4–5 cm were staged as IIA but the adjuvant therapy strategy was not changed. As stage IB–IIA LUAD patients form a highly heterogeneous group, the use of tumor size to differentiate outcomes of patients is not accurate. Some researchers have investigated the prognostic value of risk factors and developed nomograms. A previous study mentioned that VI is the independent prognostic factor of stage I NSCLC (23). Another one reported that the tumor histological grade is associated with OS in stage I NSCLC (24). Tu et al. reviewed 4,238 stage IB patients without VPI and VI, and they identified eight factors associated with lung cancer-specific survival and constructed nomograms based on them (25). Zou et al. reviewed 5,513 patients from the SEER database and 440 patients from a single center, and they identified six variants as independent prognostic factors of OS, and developed a nomogram based on them (20). Considering the prognostic value of GGO in stage IB–IIA LUAD, we first established the nomogram integrating pathological tumor size, at least 10 LNs resection, and CTR ≥0.75 to predict individual OS and DFS for pathological stage IB–IIA LUAD patients. Our nomograms showed satisfactory predictive performance with an excellent Harrell’s C-index for OS (0.660; 95% CI 0.598–0.721) and DFS (0.634; 95% CI 0.588–0.690), and the DCA showed net benefit can be acquired for our nomograms.

Several studies have shown contradictory results for ACT in pathological stage IB–IIA NSCLC. Morgensztern et al. analyzed the data of 25,267 patients with T2N0M0 NSCLC from the National Cancer Data Base (NCDB) and showed that ACT was the positive prognostic factor in OS (26). In contrast, the large RCT, CALGB 9633 trial, denied the positive effect of ACT in stage IB–IIA patients (18). In addition, Li et al. used propensity score matching to control bias and found that stage IB patients after radical resection did not benefit from ACT (27). It should be noted that the study of Li et al. ignored the heterogeneity in stage IB patients. The analysis of different subgroups in the CALGB 9633 trial revealed that patients with larger than 4 cm tumors can benefit from ACT. Increasing researchers are acknowledging the heterogeneity in pathological stage IB–IIA patients and working to identify the subgroup of patients that may benefit from ACT. Wang et al. reviewed 2,633 stage I patients from a single canter and demonstrated that VI can be used as an indicator to select suitable patients for ACT (19). Qian et al. retrospectively analyzed 1,131 patients with pathological stage IB LUAD and showed that ACT prolonged the survival in solid/micropapillary pattern subgroup (28). A previous study focused on stage IB–IIA NSCLC and reported that ACT improved the OS of patients with VPI (29). Another study used the SEER database and found that stage IB patients with poor differentiation can benefit from ACT (20). A multicenter retrospective study included 3,346 stage I LUADs and suggested stage IB patients with spread through air spaces (STAS) for ACT (30). Personalized chemotherapy based on a comprehensive assessment of the risk of recurrence is undoubtedly a better therapeutic strategy. In this study, ACT was not associated with improved survival in the entire stage IB patient cohort, but ACT was found to be a positive prognostic factor in patients with nomogram total point ≥5 based on the nomogram for OS.

There are some limitations to this study. First, this study is a retrospective single-center study and did not involve external validation, therefore, selection bias and recall bias were inevitable. In addition, the sample size in our study was limited. To ensure statistical efficiency, we did not randomly divide patients into training and validation cohorts. Therefore, these two nomograms should practically be used with caution, and we expect a large sample of external independent data to further validate our model in the future. Moreover, some factors such as STAS have been recognized as the negative prognostic factor for NSCLC, which did not enroll in our nomogram.

In conclusion, a small proportion of GGO component cannot improve OS in patients with pathological stage IB–IIA LUAD, but CTR <0.75 is associated with better DFS. Our nomogram developed by integrating pathological tumor size, at least 10 LNs resection, and CTR ≥0.75 for predicting individual OS and DFS displayed relatively good predictive capability and clinical value. The prognostic nomogram could serve as a useful tool for identifying candidate patients benefiting from ACT.

## Data Availability Statement

The datasets presented in this study can be found in online repositories. The names of the repository/repositories and accession number(s) can be found below: the Research Data Deposit (http://www.researchdata.org.cn), with the Approval RDD number: RDDA2022652259.

## Ethics Statement

The studies involving human participants were reviewed and approved by the Institutional Review Board of the Sun Yat-sen University Cancer Center. Written informed consent for participation was not required for this study in accordance with the national legislation and the institutional requirements.

## Author Contributions

Conception and design: JW and SQ. Provision of study materials or patients: WZ, GL, and YZ. Collection and assembly of data: QY, RL, DL, and WW. Data analysis and interpretation: WZ, GL, and YZ. Manuscript writing and editing: WZ, GL, and YZ. All authors listed have made a substantial, direct, and intellectual contribution to the work and approved it for publication.

## Funding

This work was supported by the Natural Science Foundation of Guangdong Province of China (Grant Numbers 2019A1515011601, 2019A1515010298).

## Conflict of Interest

The authors declare that the research was conducted in the absence of any commercial or financial relationships that could be construed as a potential conflict of interest.

## Publisher’s Note

All claims expressed in this article are solely those of the authors and do not necessarily represent those of their affiliated organizations, or those of the publisher, the editors and the reviewers. Any product that may be evaluated in this article, or claim that may be made by its manufacturer, is not guaranteed or endorsed by the publisher.
