# Supplementary material for: Ground Glass Opacity and Adjuvant Chemotherapy in Pathological Stage IB–IIA Lung Adenocarcinoma
Source: Front Oncol. 2022 Mar 25;12:851276. doi: 10.3389/fonc.2022.851276 (PMC8990754; doi:10.3389/fonc.2022.851276)
Supplement: Supplementary file 1 [file Table_1.docx]

| Regime | Number |
| --- | --- |
| Pemetrexed + Carboplatin | 51 (40.8) |
| Pemetrexed + Cisplatin | 32 (25.6) |
| Pemetrexed + Nedaplatin | 15 (12) |
| Paclitaxel + Carboplatin | 4 (3.2) |
| Paclitaxel + Cisplatin | 1 (0.8) |
| Gemcitabine + Cisplatin | 1 (0.8) |
| Pemetrexed | 18 (14.4) |
| Cisplatin | 3 (2.4) |

**Supplementary Table 1** The details of ACT regimen
