# Supplementary material for: Ground Glass Opacity and Adjuvant Chemotherapy in Pathological Stage IB–IIA Lung Adenocarcinoma
Source: Front Oncol. 2022 Mar 25;12:851276. doi: 10.3389/fonc.2022.851276 (PMC8990754; doi:10.3389/fonc.2022.851276)
Supplement: Supplementary file 2 [file Table_2.docx]

Point assignment and prognostic score

| Variable and prognostic score | Point | Estimated 3-year survival | Estimated 3-year survival |
| --- | --- | --- | --- |
| **OS** |  |  |  |
| Pathological tumor size (cm) |  |  |  |
| 1 | 2 |  |  |
| 2 | 4 |  |  |
| 3 | 6 |  |  |
| 4 | 8 |  |  |
| 5 | 10 |  |  |
| At least 10 LNs resection |  |  |  |
| Positive | 0 |  |  |
| Negative | 6 |  |  |
| CTR |  |  |  |
| CTR<0.75 | 0 |  |  |
| CTR≥0.75 | 3 |  |  |
| Total point |  |  |  |
| 11 |  | 0.95 |  |
| 16 |  | 0.90 |  |
| 4 |  |  | 0.95 |
| 8 |  |  | 0.90 |
| 13 |  |  | 0.80 |
| 17 |  |  | 0.70 |
| **DFS** |  |  |  |
| Pathological tumor size (cm) |  |  |  |
| 1 | 2 |  |  |
| 2 | 4 |  |  |
| 3 | 6 |  |  |
| 4 | 8 |  |  |
| 5 | 10 |  |  |
| At least 10 LNs resection | 0 |  |  |
| Positive | 0 |  |  |
| Negative | 5 |  |  |
| CTR |  |  |  |
| CTR<0.75 | 0 |  |  |
| CTR≥0.75 | 4 |  |  |
| Total point |  |  |  |
| 3 |  | 0.9 |  |
| 10 |  | 0.8 |  |
| 13 |  | 0.7 |  |
| 19 |  | 0.5 |  |
| 5 |  |  | 0.8 |
| 8 |  |  | 0.7 |
| 14 |  |  | 0.5 |
| 19 |  |  | 0.3 |
| OS: overall survival, DFS: disease-free survival, CTR: consolidation-to-tumor ratio, LN: lymph node. | | | |
